# Frequency Analysis of Acoustic Data Using Multiple-Measurement Sparse Bayesian Learning

**DOI:** 10.3390/s21175827

**Published:** 2021-08-30

**Authors:** Myoungin Shin, Wooyoung Hong, Keunhwa Lee, Youngmin Choo

**Affiliations:** Department of Ocean Systems Engineering, Sejong University, Seoul 05006, Korea; myoungin@sju.ac.kr (M.S.); wyhong@sejong.ac.kr (W.H.); nasalkh2@sejong.ac.kr (K.L.)

**Keywords:** frequency analysis, sparse Bayesian learning, in-situ multiple measurements

## Abstract

Passive sonar systems are used to detect the acoustic signals that are radiated from marine objects (e.g., surface ships, submarines, etc.), and an accurate estimation of the frequency components is crucial to the target detection. In this paper, we introduce sparse Bayesian learning (SBL) for the frequency analysis after the corresponding linear system is established. Many algorithms, such as fast Fourier transform (FFT), estimate signal parameters via rotational invariance techniques (ESPRIT), and multiple signal classification (RMUSIC) has been proposed for frequency detection. However, these algorithms have limitations of low estimation resolution by insufficient signal length (FFT), required knowledge of the signal frequency component number, and performance degradation at low signal to noise ratio (ESPRIT and RMUSIC). The SBL, which reconstructs a sparse solution from the linear system using the Bayesian framework, has an advantage in frequency detection owing to high resolution from the solution sparsity. Furthermore, in order to improve the robustness of the SBL-based frequency analysis, we exploit multiple measurements over time and space domains that share common frequency components. We compare the estimation results from FFT, ESPRIT, RMUSIC, and SBL using synthetic data, which displays the superior performance of the SBL that has lower estimation errors with a higher recovery ratio. We also apply the SBL to the in-situ data with other schemes and the frequency components from the SBL are revealed as the most effective. In particular, the SBL estimation is remarkably enhanced by the multiple measurements from both space and time domains owing to remaining consistent signal frequency components while diminishing random noise frequency components.

## 1. Introduction

The passive sonar system receives underwater acoustic signals that include marine objects, such as surface ships and submarines. The signals are typically composed of narrowband and broadband components. In particular, the frequency estimation of the tonal components (extremely narrowband components) is an important issue for detecting and identifying marine objects [1,2,3].

The traditional methods for frequency estimation are based on discrete Fourier transform (DFT). Practically, a fast version DFT of fast Fourier transform (FFT) is used for rapid calculations. The FFT has a limitation of frequency resolution by a limited signal length. To enhance the resolution, various frequency estimation algorithms including the estimation of signal parameters via rotational invariance techniques (ESPRIT) [4], multiple signal classification (RMUSIC) [5], and compressive sensing (CS) [6] have been proposed.

The ESPRIT and RMUSIC estimate frequency components using a signal subspace from the correlation matrix of the acoustic signal and display super-resolution frequency detection results. However, to obtain the signal subspace reliably, a large number of observations are required. Furthermore, these algorithms have the prerequisite condition of the signal component number in the observations for accurate frequency estimation and their performances significantly deteriorate at low signal to noise ratio (SNR) [7,8,9,10].

The CS solves a linear system (generally, undetermined linear system) via sparse representation using limited observations [6]. In underwater acoustics, the CS has predominantly been applied to high-resolution beamforming and line spectral estimation [11,12,13,14,15,16,17,18]. Meanwhile, the conventional CS has a basis mismatch problem because the true values in the continuous domain are expressed using the sparse representation in the discrete domain. Atomic norm minimization (ANM) is adopted in order to mitigate the problem [19,20,21]. However, it requires a computational burden with respect to the linear system size.

Both conventional and advanced CS suffers from manually determined regularization parameters controlling the sparsity of the solution [6,11,12,13,14,15,16,17,18,19,20,21]. In order to circumvent the limitation of CS, sparse Bayesian learning (SBL) has been applied in underwater acoustics. The SBL proposed by Tipping [22] for the classification and regression is one of the sparse signal recovery approaches using Bayesian inference [23]. The hyperparameters of source and noise variances in the SBL are automatically obtained via its iterative optimization processing and this makes the SBL more useful.

Recently, the SBL has been used in finding the direction of arrivals (DOAs) [24,25,26,27,28,29,30,31,32,33,34,35], localizing acoustic sources [35,36,37,38], and mode extraction [39]. Similar to CS, the SBL suffers from the basis mismatch arising from the discrete representation in the linear system, and the off-grid SBL models using approximations are proposed in order to relieve the problem [25,26,27,28,29,30]. On the other hand, noise in the measurements degrades the SBL based beamforming performance and the SBL is expanded to enhance the performance by using multiple measurements [24,25,26,27,28,29,30,31,32,33,34,35,36,37,38,39]. In the SBL using multiple measurements, the commonality of the source signals over the multiple measurements is exploited, and it increases the robustness of the SBL to the noise. Besides, the SBL using multiple measurements has a similar computational time to that for the SBL using a single measurement [24].

Here, based on the properties demonstrated in the previous works [24,25,26,27,28,29,30,31,32,33,34,35,36,37,38,39], the SBL using multiple measurements is used for detecting frequency components corresponding to the tonal signals in the passive sonar system after the linear system for the frequency analysis is established. The sparse tonal signals and the multiple measurements along the sensors in the sonar system should allow the SBL to have obvious frequency detections. However, to the best of the authors’ knowledge, the SBL has not been applied for frequency detection. Thus, in the current study, the detection results from the SBL are investigated by comparing them to those from FFT, ESPRIT, and RMUSIC. The paper is organized as follows. Section 2 provides the signal model of the frequency analysis for the linear system. Section 3 introduces the SBL and its extension for the multiple measurements. The frequency detection results using synthetic data and in-situ data are displayed in Section 4 and Section 5, respectively, and the performances of the frequency analysis schemes are analyzed by comparison. In Section 6, a brief discussion is given. Section 7 summarizes the present study.

## 2. Signal Model for the Frequency Analysis

The signal that is received by the passive sonar system can be classified into four signal types as follows: tonal signals, propeller noise, hydrodynamic noise, and ambient noise [21,40]. The tonal signals, which are generated by the machinery component of marine objects, are generally present at low-frequency bands. The propeller noise from the cavitation is produced by the propeller rotation, and the corresponding frequency components are widely located at high-frequency bands. Hydrodynamic noise is generated by the friction between a marine object and the surrounding medium. Ambient noise includes surface noise, turbulence, and noise from the tectonic activity at the sea bottom, etc. In the current study, the tonal signals are prominent components in the passive signal after applying an analog filter (or low-pass filter) suppressing other signal types to the raw data at the passive sonar system as in the conventional digital system and are used in order to detect the marine objects [41,42,43]. The filtered signal is expressed as:

(1) y(t)=s(t)+n(t),

where s(t) is the tonal signal and n(t) is the remaining ambient noise after the low-pass filtering. The tonal signal s(t) is decomposed using Fourier transform (FT) as follows:

(2) $$s\left(t\right)=\int_{-\infty }^{\infty }S\left(f\right)e^{+j2\pi ft}df,$$

where S(f) is the frequency response of s(t) at a frequency f. Since the raw acoustic data passes through the analog filter that suppresses the signals at high frequencies and prevents aliasing, low-frequency components between $-f_{s}/2$ and $+f_{s}/2$ dominantly remain, and the infinite integral can be replaced by the finite integral in the range including the remaining frequency band ($f_{s}$ is a sampling frequency of the given digital sonar system). After sampling both in time and frequency domains, Equation (2) is expressed in the discretized domains as follows:

(3) $$s\left(n\Delta t\right)=s\left[n\right]\approx \overset{M-1}{\underset{m=0}{\sum }}S_{m}e^{+j2\pi \left(m\Delta f\right)\left(n\Delta t\right)}\Delta f,$$

where $S_{m}=S(m\Delta f$), $\Delta f=f_{s}/M$, and $\Delta t=1/f_{s}$. M is an integer that determines the frequency resolution Δf, which must be greater than or equal to the number of the discrete-time signal N [40,41]. The summation corresponds to the integral from 0 to $f_{s}$, which is equivalent to the previously mentioned finite integral after the filtering owing to the periodicity $f_{s}$ of the FT of the discretized signal s(nΔt). Then, the discrete-time signal including the noise is represented in the form of vector and matrix as follows:

(4) $$\left(\begin{array}{c} y\left[0\right]\\ \vdots \\ y\left[N-1\right] \end{array}\right)=\left(\begin{array}{ccc} e^{+j2\pi \frac{0\cdot 0}{M}} & \cdots & e^{+j2\pi \frac{\left(M-1\right)\cdot 1}{M}}\\ \vdots & \ddots & \vdots \\ e^{+j2\pi \frac{0\cdot \left(N-1\right)}{M}} & \cdots & e^{+j2\pi \frac{\left(M-1\right)\cdot \left(N-1\right)}{M}} \end{array}\right)\left(\begin{array}{c} S_{0}\Delta f\\ \vdots \\ S_{M-1}\Delta f \end{array}\right)+\left(\begin{array}{c} n\left[0\right]\\ \vdots \\ n\left[N-1\right] \end{array}\right).$$

Equation (4) is rearranged by y=Ax+n, where y, A, x, and n are the measured data (the vector on the left side of the equation), the dictionary matrix (the matrix on the right side of the equation), the unknown which is relevant to the frequency component amplitude (the vector multiplied by the dictionary matrix), and the noise (the last term in the equation), respectively.

The time and frequency resolutions determine the numbers of rows (N) and columns (M) in the dictionary matrix, respectively. Particularly, when M equals N, the dictionary matrix turns into a DFT matrix, and the frequency components can be obtained by multiplying the inverse of the DFT matrix to Equation (4), which is equivalent to the frequency analysis using the FFT. When using the FFT, a large signal length from a long-time signal is required in order to increase the frequency resolution, and the frequency detection results are inevitably contaminated by the noise.

In order to enhance the frequency resolution without a longer measurement, M must be much larger than N, and Equation (4) becomes an underdetermined linear system, which should have infinite solutions. In the passive sonar system, the tonal signals have sparse frequency components, and the underdetermined linear system can be solved by exploiting the sparsity of the unknown comprising the frequency components. Thus, we apply the SBL (described in the following section), which recovers the sparse solution from the linear system using the Bayesian framework.

## 3. Introduction of Sparse Bayesian Learning

CS is one of the representative signal processing techniques that solves the underdetermined linear system using sparse signal reconstruction. The sparsity condition is explicitly imposed on unknown x in the CS [6,11,12]. Since the tonal signals are composed of the sparse frequency components at low-frequency bands, CS can be applied to the filtered signal in order to estimate the frequency components. However, when using CS, a regularization parameter, which simultaneously influences the fitness of the solution to measurement and the sparsity of the solution, is determined manually, and computational burden is relative to the number of multiple measurements. In the current study, in order to overcome the problems in the CS, we apply the SBL [22] using the Bayesian inference to the passive sonar frequency analysis, which results in high-resolution frequency estimations with less noise due to the sparsity of the SBL solution (shown in Section 4 and Section 5).

### 3.1. Single-Measurement SBL

In the SBL framework, unknown x and noise n are treated as random variables and are assumed to follow zero-mean Gaussian distributions, and the SBL solves the linear system of Equation (4) using the given measurement y by finding $\hat{x}$ which maximizes the probability as follows:

(5) $$\hat{x}=\underset{x}{\mathrm{argmax}}\;p\left(x,\gamma ,\sigma^{2}|y\right)$$

(6) $$=\underset{x}{\mathrm{argmax}}\;p\left(x|y,\gamma ,\sigma^{2}\right)p(\gamma ,\sigma^{2}|y),$$

where γ is the variance of the unknown, and $\sigma^{2}$ is the variances of noise, and they are obtained from y via maximizing $p\left(\gamma ,\sigma^{2}|y\right)$ (the second term of Equation (6)), which is equivalent to $p\left(y|\gamma ,\sigma^{2}\right)$ when the variances follow uniform distributions as in the current study [22,23,24]. Subsequently, the solution $\hat{x}$ is derived with a maximum a posteriori (MAP) estimate of $p\left(x|y,\gamma ,\sigma^{2}\right)$ (the first term of Equation (6)) using previously estimated γ and $\sigma^{2}$ with y.

Probability models in the SBL, which are used for the estimations, are introduced as follows. The posterior probability distribution $p\left(x|y,\gamma ,\sigma^{2}\right)$ can be denoted as in Equation (7) using Bayes’ theorem:

(7) $$p\left(x|y,\gamma ,\sigma^{2}\right)=\frac{p\left(y|x,\sigma^{2}\right)p\left(x|\gamma \right)}{p(y|\gamma ,\sigma^{2})},$$

where $p\left(y|x,\sigma^{2}\right)$ and p(x|γ) are the likelihood function and prior function, respectively. The denominator $p\left(y|\gamma ,\sigma^{2}\right)$ is the evidence (marginal likelihood) used to evaluate the hidden variables (or hyperparameters) of the variances.

The likelihood function $p\left(y|x,\sigma^{2}\right)$ is a Gaussian probability distribution, which arises from the linear system, whose noise following the zero-mean Gaussian distribution having the constant variance of $\sigma^{2}$ is expressed as follows:

(8) $$p\left(y|x,\sigma^{2}\right)=\frac{1}{{\left(\pi \sigma^{2}\right)}^{N}}\exp\left(-\sigma^{-2}∥y-Ax{∥}^{2}\right).$$

The unknown x is composed of an element $x_{m}$ following the zero-mean Gaussian distribution with the variance $\gamma_{m}$ (element of γ), and the prior probability is denoted as follows:

(9) $$p\left(x|\gamma \right)=\overset{M}{\underset{m=1}{\prod }}p\left(x_{m};0,\gamma_{m}\right)=\frac{1}{\pi^{N}\prod_{m=1}^{M}\gamma_{m}}\exp\left(-\Sigma_{m=1}^{M}\gamma_{m}^{-1}x_{m}^{2}\right).$$

In the SBL, the components of x are activated when the corresponding components of γ have non-zero values. During the iterative estimation in the SBL, γ turns into a sparse vector, which is an advantage for the high-resolution estimation as well as the denoising. 

The evidence is the integration of the product of the likelihood function and the prior probability distribution over x, and is expressed as follows:

(10) $$p\left(y|\gamma ,\sigma^{2}\right)=\int p\left(y|x,\sigma^{2}\right)p\left(x|\gamma \right)dx=\frac{1}{\pi^{N}\det\Sigma_{y}}\exp\left(-y^{H}\Sigma_{y}^{-1}y\right),$$

where $\Sigma_{y}=\sigma^{2}I_{N}+A\Gamma A^{H}$ (Γ=diag(γ)).

By inserting Equations (8)–(10) into Equation (7), we can get the posterior probability distribution as follows:

(11) $$p\left(x|y,\gamma ,\sigma^{2}\right)=\frac{1}{\pi^{N}\Sigma_{x}}\exp\left({\left(x-\mu_{x}\right)}^{H}\Sigma_{x}^{-1}\;\left(x-\mu_{x}\right)\right),$$

where $\mu_{x}=\Gamma A^{H}\Sigma_{y}^{-1}y$ and $\Sigma_{x}={\left(\sigma^{-2}A^{H}A+\Gamma^{-1}\right)}^{-1}$. Note that the posterior probability distribution has a maximum at $\mu_{x},$ which is the solution of the linear system, such as $\hat{x}=\mu_{x}$.

In order to derive the solution, the variances (γ and $\sigma^{2}$) are required with the measurement y, which are obtained using the probability model $p\left(y|\gamma ,\sigma^{2}\right)$ that was previously mentioned.

(12) $$\left(\hat{\gamma },{\hat{\sigma }}^{2}\right)=\underset{\gamma ,\sigma^{2}}{\mathrm{argmax}}\;p\left(y|\gamma ,\sigma^{2}\right).$$

That means that we estimate the hyperparameters of $\hat{\gamma }$ and ${\hat{\sigma }}^{2}$ using a type-II maximum likelihood.

A log-likelihood form is employed for the hyperparameter estimation. $\hat{\gamma }$ is the vector that satisfies Equation (13).

(13) $$\hat{\gamma }=\underset{\gamma }{\mathrm{argmax}}\;\log p(y|\gamma ,\sigma^{2})\propto \underset{\gamma }{\mathrm{argmax}}\left(-y^{H}\Sigma_{y}^{-1}y-\log\det\Sigma_{y}\right).$$

The derivative of Equation (13) for finding the maximum provides the update rule for $\gamma_{m}$ during the iterative estimation in the SBL as follows [24]:

(14) $$\gamma_{m}^{new}=\gamma_{m}^{old}\frac{∥y^{H}\Sigma_{y}^{-1}a_{m}∥{}_{2}^{2}}{a_{m}^{H}\Sigma_{y}^{-1}a_{m}},$$

where $\gamma_{m}^{new}$ and $\gamma_{m}^{old}$ are present and previous values for $\gamma_{m}$, and $a_{m}$ is the mth column of the dictionary matrix A. ${\left(m\right)}^{H}$ is the Hermitian transpose of vector m.

In order to estimate the noise variance ${\hat{\sigma }}^{2},$ the expectation-maximization (EM) algorithm has been used [22,23]. Meanwhile, in [24], the authors applied a stochastic maximum likelihood, which estimates the noise variance asymptotically and efficiently, and the update rule for $\sigma^{2}$ using the stochastic maximum likelihood is denoted as follows:

(15) $${\left(\sigma^{2}\right)}^{new}=\frac{1}{N-K}\left(I_{N}-A_{M}A_{M}^{+}\right)yy^{H}$$

Here, Equation (15) is used for the noise variance evaluation when a single measurement is given. The active set $M=\left\{m\in \mathbb{N}|K\;\mathrm{largest}\;\mathrm{peaks}\;\mathrm{in}\;\gamma^{new}\right\}$. $A_{M}$ is the matrix that is formed by K columns of A, which are indexed by M, and $A_{M}^{+}$ is the Moore-Penrose pseudo-inverse of $A_{M}$ [24,44]. While K must be predefined manually before the iteration, it is insensitive to the solution $\hat{x}$.

### 3.2. Multiple-Measurement SBL

During capturing passive sonar data, multiple sensors composed of an array in the sonar system are used and are kept for recording the passive sounds. Thus, the multiple measurements from two different domains (space and time) are available for frequency analysis. Basically, an average of the separate estimations according to a single measurement belonging to the multiple measurements can be used as the final evaluation of frequency components. However, here, common signal frequency components over the multiple measurements are exploited for the SBL estimation, where signal and noise variances are consistent with the measurements, to increase robustness to noise as in [24,33,34].

The multiple measurements posterior probability distribution $p\left(X|Y,\gamma ,\sigma^{2}\right)$ can be denoted as follows:

(16) $$p\left(X|Y,\gamma ,\sigma^{2}\right)=\frac{p\left(Y|X,\sigma^{2}\right)p\left(X|\gamma \right)}{p(Y|\gamma ,\sigma^{2})},$$

where Y is the N×L measurement matrix comprising measurements ($y_{l}$) as its columns, X is the M×L unknown matrix (*l*th column is the unknown vector ($x_{l}$) relevant to $y_{l}$), and N is the N×L noise matrix (*l*th column is noise ($n_{l}$) in $y_{l}$). L is the total number of multiple measurements.

In the SBL using multiple measurements, $x_{l}$ and $n_{l}$ are assumed independent across snapshots and sensors. Then, the posterior probability can be represented by $p\left(X|Y,\gamma ,\sigma^{2}\right)=p\left(x_{1}|y_{1},\gamma ,\sigma^{2}\right)p\left(x_{2}|y_{2},\gamma ,\sigma^{2}\right)\cdots p\left(x_{L}|y_{L},\gamma ,\sigma^{2}\right)$. Then, we can express the likelihood function, prior probability, and evidence for the multiple measurements using the product of each single measurement probability as follows:

(17) $$p\left(Y|X,\sigma^{2}\right)=\overset{L}{\underset{l=1}{\prod }}\frac{1}{{\left(\pi \sigma^{2}\right)}^{N}}\exp\left(-\sigma^{-2}∥y_{l}-Ax_{l}∥{}^{2}\right).$$

(18) $$p\left(X|\gamma \right)=\overset{L}{\underset{l=1}{\prod }}\frac{1}{\pi^{N}\prod_{m=1}^{M}{\left(\gamma_{m}\right)}_{l}}\exp\left(-\Sigma_{m=1}^{M}{\left(\gamma_{m}^{-1}\right)}_{l}{\left({\left(x_{m}\right)}^{2}\right)}_{l}\right).$$

(19) $$p\left(Y|\gamma ,\sigma^{2}\right)=\overset{L}{\underset{l=1}{\prod }}\frac{1}{\pi^{N}\det\Sigma_{y_{l}}}\exp\left(-y_{l}^{H}\Sigma_{y_{l}}^{-1}y_{l}\right).$$

By inserting Equations (17)–(19) into Equation (16), the posterior probability for the multiple measurements is expressed by Equation (20).

(20) $$p\left(X|Y,\gamma ,\sigma^{2}\right)=\overset{L}{\underset{l=1}{\prod }}\frac{\exp\left({\left(x_{l}-\mu_{x_{l}}\right)}^{H}\Sigma_{x_{l}}^{-1}\;\left(x_{l}-\mu_{x_{l}}\right)\right)}{\pi^{N}\Sigma_{x_{l}}},$$

where $\mu_{x_{l}}$ is the lth column of $\mu_{X}$, and $\mu_{X}=\Gamma A^{H}\Sigma_{y}^{-1}Y$. $\mu_{X}$ is the solution of the linear system Y=AX+N (i.e., $\hat{X}=\mu_{X}$), which are averaged over the columns (or measurements) for the final estimation.

For the solution, we estimate the hyperparameter $\hat{\gamma }$ and ${\hat{\sigma }}^{2}$ by the type-II maximum likelihood. Through the same process as in the single-measurement SBL, the update rules for the common variances along the measurements are derived as follows:

(21) $$\gamma_{m}^{new}=\frac{\gamma_{m}^{old}}{L}\overset{L}{\underset{l=1}{\sum }}∥y_{l}^{H}\Sigma_{y}^{-1}a_{m}∥{}_{2}^{2}/a_{m}^{H}\Sigma_{y}^{-1}a_{m}.$$

(22) $${\left(\sigma^{2}\right)}^{new}=\frac{1}{L\left(N-K\right)}\overset{L}{\underset{l=1}{\sum }}\left(I_{N}-A_{M}A_{M}^{+}\right)y_{l}y_{l}^{H}.$$

As shown in Equations (21) and (22), the averages of source and noise variances along the multiple measurements are used for the update during the iterative process in the SBL. The multiple-measurement SBL has advantages in robust estimation owing to the averages enhancing the common signal components while diminishing the random noise components.

## 4. Frequency Analysis Using Synthetic Data

In this section, we present the frequency detection results using synthetic data, which allows performance analysis of the SBL according to various SNRs and frequency component numbers. For the comparison, we applied FFT, ESPRIT, and RMUSIC, which are the representative frequency analysis algorithms, along with the SBL. The frequency detection performance is compared in terms of the recovery ratio ($R_{c}$), which is defined as the ratio of restored frequency component number to the total frequency component number (see Equation (23)).

We generate K tonal signals for the synthetic data, where the tonal frequency components are chosen randomly in the range from 0–500 Hz, and the corresponding amplitudes are from the Gaussian distribution of N(1,0.1). For the frequency analysis, 25 multiple measurements of 0.2 s-length signals sampled with 1 kHz (twice the highest frequency) are used. The signal length determines the FFT frequency resolution, and the signal frequency components within 5 Hz (inverse of 0.2 s) cannot be resolved by the FFT owing to the insufficient signal length. When using the SBL for the frequency estimation, the frequency difference between adjacent columns is set as 1 Hz in order to improve the frequency resolution with the same measurements, and it induces the underdetermined linear system of Equation (4) as expected. The zero-mean white Gaussian noise $n_{l}$ is added to clean signal s, and the corresponding SNR is computed as follows [21,24,33,34]: SNR $=10\log\left({∥s∥}_{2}^{2}/\frac{1}{L}\sum_{l=1}^{L}∥n_{l}∥{}_{2}^{2}\right)$.

**Figure 1:**
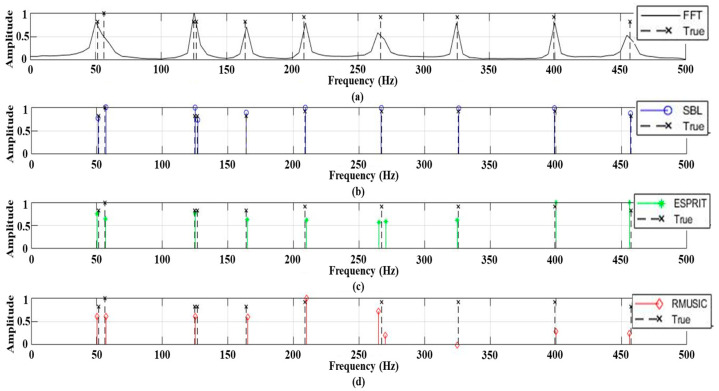
The frequency estimation results using (**a**) fast Fourier transform (FFT); (**b**) sparse Bayesian learning (SBL); (**c**) estimation of signal parameter via rotational invariance techniques (ESPRIT); (**d**) root multiple signal classification (RMUSIC). The signal includes ten frequency components (marked by x) at an SNR of 15 dB.

Figure 1 displays an example of the frequency detection results for signals including ten tonal frequency components at an SNR of 15 dB; the results are normalized by the corresponding maxima for the convenient comparison. Here, the built-in functions of *rootmusic* and *esprit* in MATLAB are used for RMUSIC and ESPRIT (Figure 1c,d), respectively, and their prerequisite condition of signal frequency component number is exactly determined in the simulation. The FFT merges the two frequency components in close proximity owing to its low resolution arising from the short signal length. While the advanced schemes of ESPRIT and RMUSIC demonstrate a better distinction of the close frequency components (frequency components around 50 Hz), they not only overlook some frequency components (frequency components around 125 Hz) but also divide a single frequency component into two (frequency component around 260 Hz). The problems are remarkably mitigated by the SBL, which reconstructs all of the signal components near the corresponding frequencies from the measurements. This is attributed to its sparse solution directly derived from the linear system and the multiple measurements which remain as consistent signal components while suppressing random noise components during the iterative process in the SBL. Meanwhile, the SBL estimations slightly deviate from the true values because the off-grid true frequency components are represented by their neighbor on-grid columns in the matrix of the linear system (basis mismatch) [18,25,26,27,28,29,30].

For the simulation in Figure 1, the FFT, RMUSIC, ESPRIT, and SBL require computational times of 0.0005 s, 0.1344 s, 0.0970 s, and 1.896 s, respectively, under the computational environment of Intel(R) Core (TM) i9-9900K CPU. Meanwhile, the modified CS of ANM recovers the frequency components from the linear system with a computational time of 544 s (not shown in the current study). Although the SBL has more computational complexity than the classical approaches because of solving the linear system directly, it is still useful in the frequency analysis owing to its ability to recover signal frequencies in high resolution and reduce false detections with moderate computational time (much less than that for CS).

We examine the frequency analysis schemes according to SNRs at a fixed frequency component number and frequency component numbers at a fixed SNR, and compare their performances in terms of the recovery ratio defined as follows:

(23) $$R_{c}=\frac{1}{C}\sum_{c=1}^{C}\frac{\sum_{i=1}^{K}{\left(r_{i}\right)}_{c}}{K}\;\mathrm{where}\;r_{i}=\{\begin{array}{c} 1,\;\;\;\left|f_{i}-{\hat{f}}_{i}\right|\leq \Delta f/2\\ 0,\;\;\;\;\;\;\;\;\;\;\;\;\;\;else\;\;\;\;\;\;\;\;\;\;\;\; \end{array},$$

where C is the number of trials (here, trials, where K tonal frequency components are randomly selected from 0 Hz to 500 Hz, are repeated 1000 times for each case), $f_{i}$ is the ith true frequency component, and ${\hat{f}}_{i}$ is the estimated frequency component corresponding to $f_{i}$. In the current study, Δf is fixed at 5 Hz, which corresponds to the frequency resolution of the FFT. Here, for a strict calculation of the recovery ratio, the *K*th strongest components from the estimation are used. That is, the refined frequency components are used to calculate the recovery ratios in trials for a specific case (e.g., certain SNR at a fixed frequency component number or certain frequency component numbers at a fixed SNR), and their mean is the represented recovery ratio at the corresponding case. The recovery ratio increases as estimated dominant peaks locate near the true frequency components, and the opposite happens when peaks from noise are significant in the estimation and those corresponding to the true frequency components are excluded in calculating the recovery ratio.

**Figure 2:**
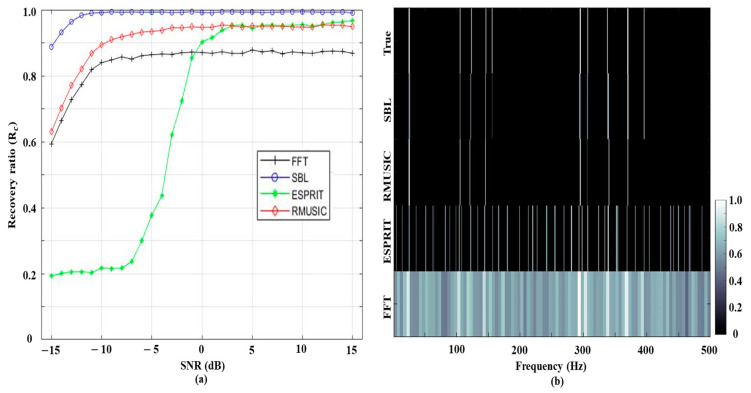
(**a**) The recovery ratios for FFT, SBL, ESPRIT, and RMUSIC according to the SNRs. Ten frequency components are included in each trial; (**b**) the detection results at the lowest SNR of −15 dB, which qualitatively displays the detected frequency components from the schemes.

The recovery ratios according to SNRs (the frequency component number is fixed at ten) are displayed in Figure 2a, where the SBL demonstrates the best performance for the overall SNRs. The noise in signals directly contaminates the frequency detections from the FFT, which deteriorate with the decrement of SNRs. While the RMUSIC and ESPRIT ameliorate the FFT detection results, their performances (in particular, ESPRIT) are significantly diminished by the intensive noise such as the beamforming [7,8,9,10]. However, the SBL has an acceptable recovery ratio of 0.88 even at the lowest SNR of −15 dB by exploiting the commonality over the multiple measurements (i.e., sharing the frequency components across the measurements). Figure 2b shows an example of frequency detections at the worst case (SNR of −15 dB), which allows a qualitative inspection of the schemes; for the comparison convenience, a 2-D contour plot with grayscale is used. In the FFT result, the intensive noise smears the true frequency components and makes the frequency analysis ambiguous. While the ESPRIT estimates the frequency components in a higher resolution, a significant number of incorrect detections across the frequency are induced by the noise. The RMUSIC diminishes the false estimations at the cost of true signal component loss. As in the high SNR simulation of Figure 1, the problems in the classical approaches for frequency analysis are alleviated by the SBL. Whereas the SBL detections have deviations from the true values by the basis mismatch and the noise lessens estimation amplitudes (in particular, those corresponding to weak frequency components), all of the tonal signals are recovered near the true values owing to the sparse evaluation using the sufficient measurements.

**Figure 3:**
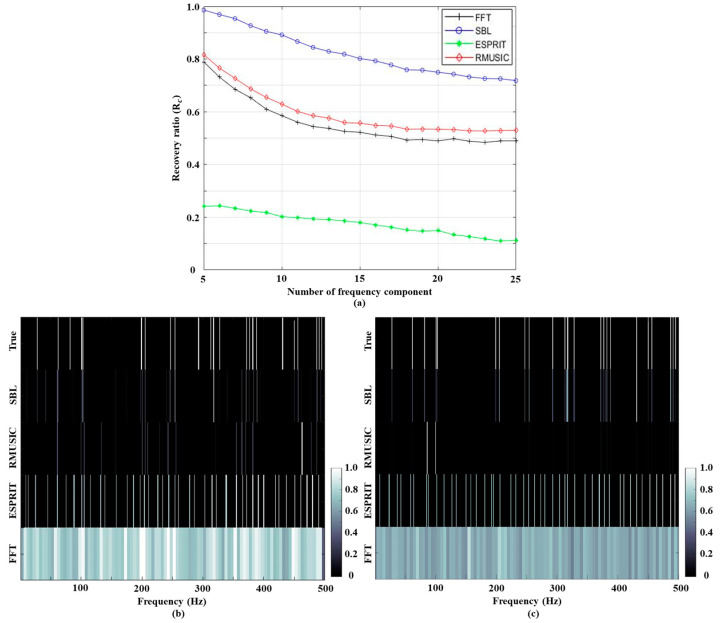
(**a**) The recovery ratios for FFT, SBL, ESPRIT, and RMUSIC according to the frequency component numbers. SNRs for the simulations are fixed at −15 dB; (**b**) the detection results for data including 25 frequency components. Signal length and measurement number are 0.2 s and 25, respectively; (**c**) the detection results for data including 25 frequency components. The signal length and measurement number increase to 0.5 s and 50, respectively, and they improve the SBL performance noticeably.

Subsequently, the recovery ratios from the schemes are investigated along with the frequency component numbers at the lowest SNR of −15 dB as shown in Figure 3a. As expected, a lesser frequency component number is beneficial for accurate frequency detections, and the recovery ratios decrease with the increment of the component number except for the ESPRIT with poor performance at low SNR as in [7,8,9,10]. As shown in Figure 3b, where 25 tonal components are included, the contaminated result from the FFT with the low resolution cannot accurately resolve the true components owing to the closer components. The denser frequency components are also detrimental to the frequency detections from ESPRIT and RMUSIC. Particularly, the number of correctly detected components from RMUSIC is reduced and the corresponding components appear faintly. Although the SBL displays the best detections among the schemes utilizing the component numbers, the sparse solution from SBL degrades the estimations relative to the component numbers. The false detections and the true component loss are improved by extra measurements holding longer signal lengths, as shown in Figure 3c, where 50 multiple measurements of 0.5 s-length signals are used for detecting 25 frequency components in Figure 3b. The SBL performance is noticeably enhanced because the longer observations provide additional equations for the underdetermined linear system (N gets bigger) and enable the SBL solution to accommodate more frequency components. Furthermore, the extra measurements reduce the previous false detections. This fact is also confirmed by the in-situ data in the following section.

## 5. Frequency Analysis Using the Underwater In-Situ Data

### 5.1. Signals in the Experiment

We present frequency analysis results for the in-situ data measured near the Korean peninsula. Tonal signals consisting of 21 frequency components including one pilot signal are transmitted from a transducer towed by an experimental ship at a speed of approximately 1 m/s–1.5 m/s. The acoustic data are recorded for 66 min by a horizontal line array (HLA) on the sea bottom, which is composed of 120 sensors; it is not allowed to disclose the operating frequency range as well as sampling frequency owing to the sonar system developed for defense, and the detection results are normalized by the maximum frequency of $f_{s}/2$.

**Figure 4:**
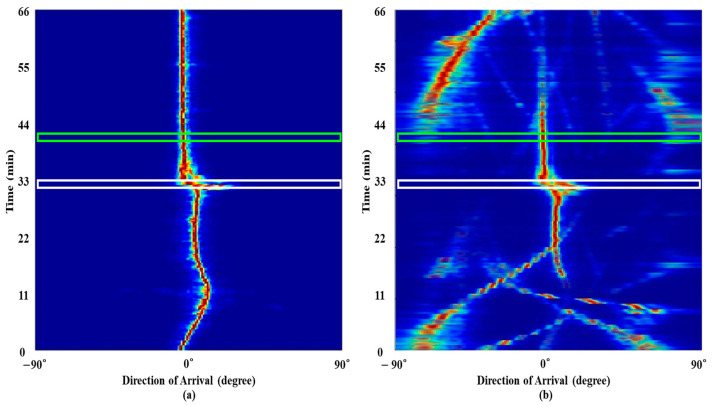
The ship tracks from conventional beamforming results using (**a**) the pilot frequency; (**b**) the frequency components from the fishing boats and the experimental ship.

**Figure 5:**
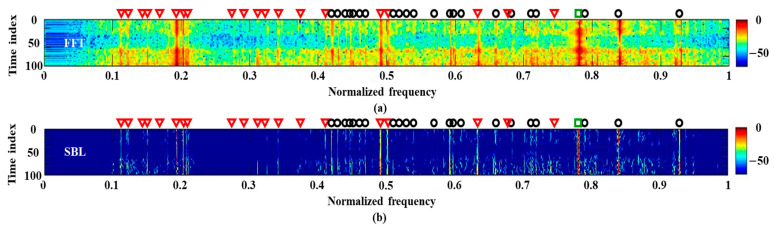
The frequency detection results using a single measurement, which is measured when the experimental ship is around the array: (**a**) FFT; (**b**) SBL.

The pilot signal has a 10 dB to 45 dB higher amplitude than the others, and the weak signals are masked by the ocean ambient noise even when multiple measurements along time and space are used. Beamforming using the strongest signal (i.e., beamforming at the frequency corresponding to the pilot signal) indicates the track of the experimental ship (Figure 4a), which approaches the array from its broadside and gets further away after 33 min; the path is confirmed by the GPS of the experimental ship. During the measurements, several fishing boats are near the array and low-frequency tonal sounds from the boats are inevitably in the recording. In Figure 4b, broadband beamforming using some passive signal components from the fishing boats and the experimental ship (marked with black circles in Figure 5), which are identified by the SBL in the following subsection, display regular tracks including the experimental ship. Thus, in the current study, the tonal signals from the transducer and the ships are referred to as signals, which should be detected using frequency analysis algorithms.

### 5.2. Frequency Analysis Using a Single Measurement

We use a single measurement with a signal length of 0.5 s for frequency detection. The signals are captured by the sensor in the middle of the HLA when the experiment ship is near the sonar system (see the white box around 33 min in Figure 4). The detection results from the FFT and SBL are displayed in Figure 5; the single measurement restricts applications of ESPRIT and RMUSIC. The frequency components are detected along the observation time. That is, the frequency analysis is conducted repeatedly (100 times in this study). Here, the time window has the same size signal length (0.5 s) strides of 0.05 s for the next frequency analysis, and each result is stacked vertically in order to observe the frequency variation over time. For a convenient comparison, overall detection results are normalized with their maximum value. When using the FFT, the stacked results are equivalent to the spectrogram. As previously mentioned, besides the tonal components from the transducer (marked by the red triangles and the green square for normal and pilot components, respectively), the narrowband signals from the ships are constantly detected, which are dominant from the experimental ship (refer to the track from passive signals near the measurement). The frequency analysis using the FFT (Figure 5a) is smeared by the overall noise, which masks the true frequency components and prevents a clear frequency detection although the acoustic signals are measured by the HLA in close proximity to the experimental ship. The SBL improves the detection result in terms of the resolution and denoising as displayed in Figure 5b; the overall noise is significantly reduced by the sparse estimation, which is beneficial to the detection clarity. However, the insufficient measurement for the estimation induces intermittently emerged false detections (in particular, the lower part of Figure 5b). In order to improve the performance, multiple measurements along space and time can be exploited.

### 5.3. Frequency Analysis Using Multiple Measurements

Since the passive sonar system is composed of multiple sensors and measures underwater sounds for sufficient time, three types of multiple measurements (time domain, space domain, and time and space domain) are used for the frequency analysis. Correspondingly, the multiple measurements data Y in the SBL is defined for each case as follows:

- Case 1: $Y=\left[y_{1}^{s},y_{2}^{s},\ldots ,y_{L_{t}}^{s}\right]$;
- Case 2: $Y=\left[y_{t}^{1},y_{t}^{2},\ldots ,y_{t}^{L_{s}}\right]$;
- Case 3: $Y=\left[y_{1}^{1},y_{1}^{2},\ldots ,y_{1}^{L_{s}},y_{2}^{1},y_{2}^{2},\ldots ,y_{2}^{L_{s}},\ldots ,y_{L_{t}}^{1},y_{L_{t}}^{2},\ldots ,y_{L_{t}}^{L_{s}}\right].$

Here, $L_{t}$ and $L_{s}$ are the numbers of snapshots (in the time domain) and sensors (in the space domain), respectively. $y_{t}^{s}$ represents the tth snapshot at the sth sensor; the subscript and superscript indicate dependences of the measurement on time and space, respectively. The first (or the second) case uses multiple measurements at a fixed sensor *s* (or at a fixed time *t*) whereas all of the measurements ($L_{t}\times L_{s}$) are exploited for the frequency detections in the last case.

**Figure 6:**
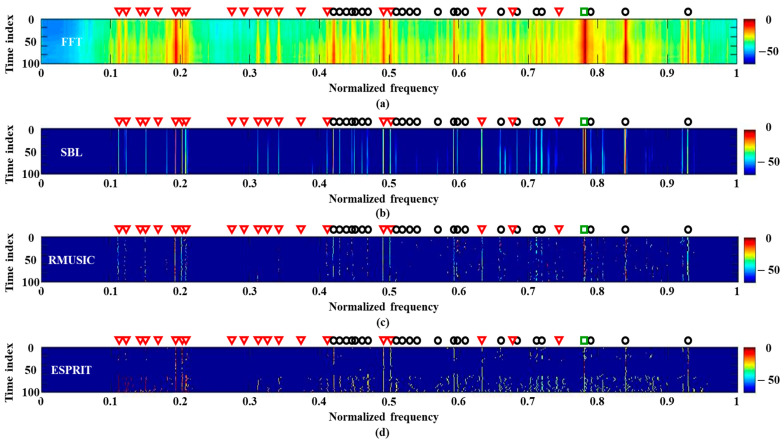
The frequency detection results using temporal multiple measurements, which are measured when the experimental ship is around the array: (**a**) FFT; (**b**) SBL; (**c**) RMUSIC; (**d**) ESPRIT.

The frequency analysis schemes are applied to 120 multiple measurements along time (case 1), which are captured by the central sensor around 33 min, and each measurement has a signal length of 0.5 s. Figure 6 displays the detection results according to the schemes including RMUSIC and ESPRIT, where the prerequisite condition of signal component number, which significantly affects the detection performances, is empirically determined as 32 (the best performances) for the in-situ data. As in the previous case, the frequency variation is observed via 100 times repeated applications of the schemes.

By using the multiple measurements, the FFT removes scattered blurs and recovers lost signal components (e.g., those between 0.3–0.4) whereas it cannot distinct adjacent signal components owing to low resolution merging the components (e.g., those around 0.2 and 0.6). While RMUSIC and ESPRIT have superior frequency resolutions, they overlook a significant number of components including those between 0.3–0.4. On the other hand, owing to exploiting the common frequency components over the measurements in the SBL, the frequency components are revealed most obviously without losing the signals, and the false detections by the noise are cleared (compare Figure 6b to Figure 5b). Estimation results using space multiple measurements (case 2) are similar to those using the temporal multiple measurements.

**Figure 7:**
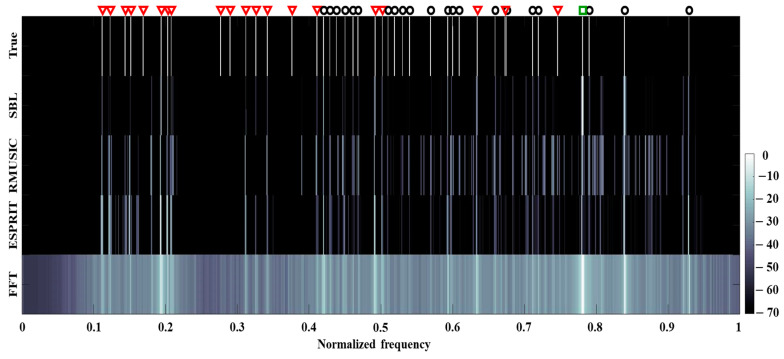
Averaged frequency detection results of Figure 6 over time according to SBL, RMUSIC, ESPRIT, and FFT. Blurs in FFT and false detections in RMUSIC and ESPRIT are mitigated by the SBL owing to its sparse estimation.

Figure 7 displays the averaged detection results of Figure 6 over time according to the SBL, RMUSIC, ESPRIT, and FFT; for comparitive convenience, the 2-D contour is plotted in the same form as Figure 2b. The signal from the in-situ data has 42 frequency components (21 tonal components from the transducer and the rest from the ships). While the signal components have different amplitudes according to the corresponding frequencies, we purposely set their amplitudes as 0 dB to highlight the locations of true frequency components. As in the simulation, the FFT suffers from overall residing blurs from the ocean noise and low resolution by the limited observation time of 0.5 s (in particular, the estimations between 0.5 and 0.6 and around 0.8). The frequency components are estimated with fewer blurs and higher resolution using the RMUSIC and ESPRIT. However, in the averaged detection results from RMUSIC and ESPRIT, significant numbers of false alarms are induced by their wobbling frequency components over time (see Figure 6c,d); that is, the inconsistent estimations over time affect the distributed estimations average. Although some frequency components from the SBL are revealed faintly or overlooked by the corresponding weak signal strengths, the consistent sparse frequency components over time remarkedly reduce the false alarms as well as the blurs. For a quantitative inspection of the performance, detected peaks within 1 Hz (the half of FFT frequency resolution) are counted for calculating the recovery ratios after selecting dominant components (here, the 42nd largest peaks of the estimations), and the numbers of perfectly matched components to the true ones are also counted according to the schemes. The recovery ratios (perfectly matched component numbers) of SBL, RMUSIC, ESPRIT, and FFT are 0.64 (23), 0.48 (7), 0.6 (13), and 0.64 (15), respectively. While the SBL has the same recovery ratio as that of the FFT, most of the components from SBL are recovered perfectly with the least false alarms, which results in the clearest frequency detection.

**Figure 8:**
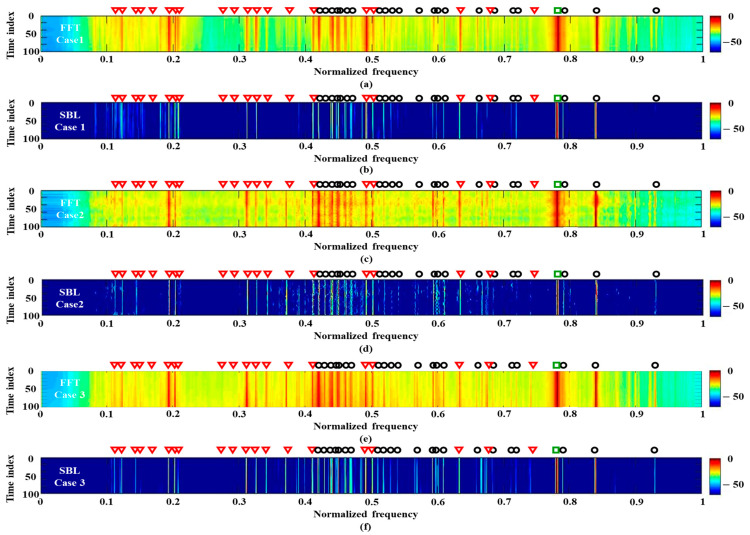
The frequency detection results using multiple measurements which are measured when the experimental ship is farther from the array: (**a**) FFT using multiple measurements over time; (**b**) SBL using multiple measurements over time; (**c**) FFT using multiple measurements across the sensors; (**d**) SBL using multiple measurements across the sensors; (**e**) FFT using multiple measurements along time and sensors; (**f**) SBL using multiple measurements along time and sensors.

Subsequently, the acoustic data captured farther from the experimental ship (the green box around 41 min in Figure 4) are used for the frequency analysis. Here, the FFT and SBL are applied to the multiple measurements in time and space domains (case 1 and case 2), separately, and the detection results are shown in Figure 8. In particular, all the acoustic signals at the sensors in the HLA are used for the spatial multiple measurements (120 multiple measurements). The frequency detections from RMUSIC and ESPRIT are too sensitive for the prior condition of the frequency component number, and they are omitted from Figure 8.

Regardless of the measurement domains, the multiple measurements reduce the ambient ocean noise. When comparing Figure 8a with Figure 8c, the measurements at the fixed sensor overlook some signal components (e.g., the components between 0.3–0.4), which might be attributed to the sensitivity of the sensor and/or the propagation loss for the sensor. Signal components missed by the sensor can be captured by other sensors in the HLA, and thus the frequency analysis using the multiple measurements along the sensors has advantages in detecting the lost signal components. However, the peak signal components from the FFT are not the same across the sensors owing to the different measurement conditions, and they induce the jitters between 0.4–0.5. As in the previous case, the SBL remarkably diminishes the ocean noise and separates the merged components in the FFT via sparse estimation using multiple measurements in time or space. The SBL result using the multiple measurements reflects the characteristics of the corresponding FFT results. While the SBL using the spatial measurements is better for detecting the frequency components, which slightly differ along the observation times and are smeared by the noise. On the other hand, the SBL using the temporal measurements achieve clean and consistent frequency components at the cost of overlooking some components.

In order to obtain the properties of both detection results, multiple measurements over both time and space domains are exploited (case 3), and 14,400 (120 × 120) multiple measurements are used for the frequency analysis. Figure 8e,f) displays the detection results using the FFT and SBL, respectively. While overall signal components (in particular, the components around 0.3 and 0.6) from the FFT appear more clearly owing to the supplement measurements, intensive components leaked from the true signals (in particular, the components between 0.4 and 0.5), as well as the low-resolution from the limited signal length, induce ambiguities in the detection. The additional measurements enhance the SBL based frequency detection more effectively. In the SBL detection results (Figure 8f), the overlooked components in Figure 8b are recovered, and the wiggling components in Figure 8d turn into the streaks with less noise. This enables the SBL with all the measurements to have the most confident frequency detection among the previously mentioned schemes.

**Figure 9:**
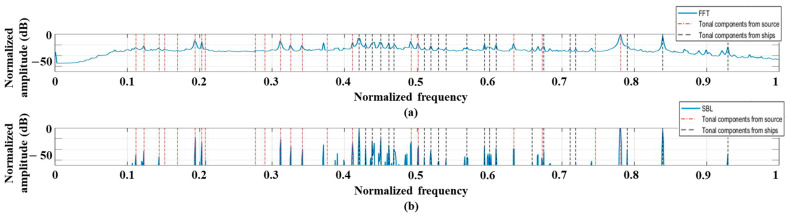
Averaged frequency detection results of Figure 8e,f over time according to (**a**) FFT and (**b**) SBL. Sparse estimations from the SBL reveal the frequency components more clearly.

Figure 9 displays the averaged frequency detection results of Figure 8e,f. As seen in Figure 9a, some frequency components from the FFT provide vague detection results because they are hidden by their neighbor noise (in particular, estimations between 0.1 and 0.2). Additionally, a minor component around 0.8 is masked by the sidelobe of the near major component, which arises from the low resolution of FFT. The lost frequency components are recovered by the SBL owing to its sparse estimations with effective exploitation of the multiple measurements (Figure 9b). The recovery ratios of FFT and SBL are the same as 0.71, respectively. However, the number of perfectly matched components from SBL (28) is much larger than that of FFT (15). Thus, the SBL is better in detecting the frequency components owing to the higher resolution with fewer false alarms.

## 6. Discussion

Here, to improve the frequency detection for the passive sonar system, the SBL is applied to the frequency analysis after establishing the corresponding linear system. The detection results using the synthetic and in-situ data are compared with those from the FFT, ESPRIT, and RMUSIC; the synthetic data are used to investigate the SBL performance in various situations, and the in-situ data allows the feasibility test of SBL. As expected, the FFT suffers from the low resolution of the limited signal length and overall blurs by the noise. Signal subspace schemes such as RMUSIC and ESPRIT recover the frequency components with higher resolution. However, their applications are limited by the prerequisite condition of signal frequency component number as well as the intensive noise [7,8,9,10]. 

The SBL solves the linear system of Equation (4) while exploiting common frequency components across multiple measurements. As demonstrated in studies using the sparse representation [24,25,26,27,28,29,30,31,32,33,34,35,36,37,38,39], the sparse estimations from the SBL using the multiple measurements have advantages for frequency detection in terms of enhancing resolution and reducing noise, which is supported by the comparison results of Section 4 and Section 5. It is noteworthy that the feasibility of SBL in detecting low-frequency components in passive sonar signals is examined using the in-situ data by the current study.

Meanwhile, the SBL has more computational complexity than those for the FFT, RMUSIC, and ESPRIT because of solving the linear system directly. Still, it is useful in frequency analysis owing to its ability of high-resolution frequency recovery and denoising with moderate computational time (much less than that for CS).

## 7. Summary

For detecting tonal signals received by the passive sonar system, we established the linear system for the frequency analysis and applied the SBL algorithm which reconstructs a sparse solution from the linear system using the Bayesian framework. The sparse estimation from the SBL enables clear frequency detections with high resolution using limited observations. In order to enhance the SBL robustness, three types of multiple measurements (time, space, and time and space) can be exploited in the passive sonar system.

We compared the frequency detection results from FFT, ESPRIT, RMUSIC, and SBL using synthetic data according to SNRs at a fixed frequency component number and frequency component numbers at a fixed SNR. The SBL displays a superior performance with lower estimation errors with a higher recovery ratio. Furthermore, the advantages of the SBL, which are recapitulated as follows, are demonstrated using the in-situ data measured near the Korean peninsula (particularly, when multiple measurements were available).

1. The overall noise is significantly reduced by the sparse estimation of the SBL, which enables a higher resolution and recovery performance than other frequency analysis algorithms such as FFT, ESPRIT, and RMUSIC;
2. The SBL using temporal multiple measurements has clean and consistent frequency component results, but it overlooked some signal components;
3. The SBL using spatial multiple measurements has advantages in detecting the lost signal components at the cost of vaguer detection results having wiggling frequency components smeared by the adjacent noise.
4. The SBL using both temporal and spatial multiple measurements has high recovery performance (advantage of the SBL using spatial multiple measurements) as well as clean and consistent frequency detections (advantage of the SBL using temporal multiple measurements).

## Data Availability

Not applicable.
